# New Insights in Potato Leaf Freezing by Infrared Thermography

**DOI:** 10.3390/app9050819

**Published:** 2019-02-26

**Authors:** Matthias Stegner, Tanja Schäfernolte, Gilbert Neuner

**Affiliations:** Department of Botany, University of Innsbruck, Innsbruck 6020, Austria

**Keywords:** differential thermal analysis, freezing susceptible, ice nucleation, ice propagation, ice tolerance

## Abstract

Infrared thermography has been widely used to study freezing processes in freezing resistant plants but hardly in freezing susceptible species. *Solanum tuberosum* leaves get frost killed at −3 °C and are unable to frost harden. The basic nature of frost injury to potato leaves is not clear. By employment of infrared differential thermal analysis (IDTA) in combination with viability assessment, we aimed to clarify the mechanistic relationship between ice formation and frost injury. During controlled freezing of potato leaves two distinct freezing events were detected by IDTA. During the first freezing event, the ice wave propagated via the xylem and spread out within 60 s throughout the whole leaf. When leaves were rewarmed after this freezing event, they did not show any frost injury symptoms. We suggest that this non-lethal first ice wave is restricted to the extracellular space. When leaves remained exposed after this exotherm, a second freezing event with a diffuse freezing pattern without a distinct starting point was recorded. When thawed after this second freezing event, leaves always showed frost damage suggesting intracellular freezing. The freezing behavior of potato leaves and its relation to frost damage corroborates that control of ice nucleation is a key for frost protection.

## 1 Introduction

After the first employments of infrared thermography to study freezing processes in plants [1–3], several advances in high-resolution infrared thermography (HRIT), brought significant new insights about ice nucleation and ice propagation in plant samples and whole individuals [4–9]. Especially, when HRIT is used in the differential imaging mode (infrared differential thermal analysis IDTA; [10–12]) ice growth in plant tissues can be visualized at such high resolution that in some cases allows for the tracing of the freezing signal to single cells in the xylem [13]. The method additionally allows the identification of plant parts or tissues that supercool and stay free of ice and usually a second freezing process upon breakdown of supercooling can be traced to intracellular lethal freezing [5,14–18]. Still, rare attempts have been made to study plant freezing under natural frost conditions by infrared thermography [6,19].

Freezing signals measured by IDTA in plant samples can originate from both extracellular or extra-tissue [20] ice formation that can be tolerated in freezing resistant plants down to a species-specific freezing temperature threshold and intracellular freezing that is usually lethal [21]. A combination of IDTA with viability tests under controlled freezing treatments permits an evaluation of the consequence that ice formation inside the tissue exhibits on cell vigor. In freezing resistant plant samples, several freezing processes at different freezing temperatures can be detected. Usually one matching frost injury is suggested to be intracellular [13,15–18]. While the overwhelming majority of studies on the freezing process in plants by infrared thermography focuses on freezing resistant plants [3,4,6,7,9–13,15–18], comparatively little information exists about plants that tolerate little or no ice [22]. Particularly, in freezing susceptible leaves the question whether ice formation per se or rather its consequences (freeze dehydration and freezing cytorrhysis) are lethal to the cells should be answerable by employment of IDTA.

*Solanum tuberosum* is a common and important field crop in the temperate regions and at high elevations in the tropics where frost is a chronic problem [23]. Unfortunately potatoes possess very little freezing resistance [24]. *S. tuberosum* leaves get frost killed around −3 °C and are unable to frost harden [25]. Under field conditions, leaf and stem tissue of potato nucleates at −0.5 to −3 °C [21,26] and plants freeze at relatively slow cooling rates, i.e., 1–3 K·h^−1^ [21]. The basic nature of frost injury at the cellular level in leaves of potatoes, and how it relates to ice formation, is still not well understood. However, important mechanisms for survival appeared to be the tolerance of freeze-induced dehydration and preservation of membrane integrity, a greater capacity to tolerate more frozen water and the avoidance of intracellular freezing [25]. In an early pioneer thermal imaging study, whole potato plants revealed a relatively high deep supercooling capacity in extreme down to −8 °C during cooling at 15 K·h^−1^ down to −10 °C [27]. However, for the investigation of how freezing processes in potato leaves relate to freezing injury, it may be advisable to keep the freezing treatment as close as possible to natural field freezing conditions. This includes control of ice nucleation, slow cooling rates (1–3 K·h^−1^) and the final target freezing temperature should not exceed the frost killing temperature of −3 °C.

We aimed to study the freezing process in potato leaves by IDTA in combination with a viability assessment in order to find out whether *S. tuberosum* leaves tolerate ice or get immediately killed upon ice formation. This should allow addressing the question of the basic nature of frost injury in *S. tuberosum* leaves. The results may have consequences for potential future means allowing frost protection in this important crop plant.

## 2 Materials and Methods

### 2.1 Plant Material

Freezing processes were studied in leaves of *Solanum tuberosum* (var. Agria Bio). During the summer season of 2018 (May till August) potato plants were cultivated in a field plot (2 m^2^) of the Botanical Garden of the University of Innsbruck (47°16′5″ N, 11°22′52″ E, 610 m a.s.l.). The field plot contained natural soil (sandy silt) with modest compost fertilization. Plants were regularly watered. Sampling of leaves was started right after the maximum canopy height had been reached. Only young but fully expanded leaves from the canopy surface were used for the experiments. Leaves were selected randomly and always detached in the morning before experiments were performed. The leaf petiole was immediately submersed and cut under water before the transport to the laboratory, which lasted less than 3 min. The leaves remained well watered till the onset of the experiments.

Air temperatures were recorded by a TidBit data logger (Onset, Bourne, MA, USA). Monthly mean/minimum temperatures were in May 17.2/8.3 °C, in June 18.7/9.6 °C, in July 20.5/11.4 °C and in August 20.9/6.1 °C.

### 2.2 Freezing Treatments

Controlled exposure to subzero temperatures of *S. tuberosum* leaves was conducted inside the cooling compartment of temperature-controlled freezers as described previously in detail [16]. As cooling rates are critical for frost injury [21], in all freezing experiments leaves were cooled at a moderate rate of 3 K·h^−1^ to target freezing temperatures. As the temperature of ice nucleation also has effects on freezing injury in potato leaves [21], ice nucleation was controlled by use of INA (ice nucleation active) bacteria (*Pseudomonas syringae* van Hall 1902) which occurred at a mean temperature of −2.3 °C ± 0.4 SE. In all freezing experiments leaf temperatures were monitored via copper constantan thermocouples (TT-TI-36, Omega, Stamford, CT, USA) that were connected to a data logger (CR10, Campbell Scientific, Logan, UT, USA).

### 2.3 Detection of Freezing Exotherms by DTA

Freezing exotherms in leaves of *S. tuberosum* were measured by the established method of differential thermal analysis (DTA; [28]). During controlled freezing treatments, leaf temperatures were monitored via copper constantan thermocouples. For detecting ice formation based on a rise in temperature due to phase-transition-heat-release, thermocouples were sandwiched between Styrofoam and the leaves. A thermocouple positioned in close vicinity to the leaves was used as a reference temperature. For the DTA-plot the differential temperature (leaf – reference temperature in K) was plotted against time. DTA was conducted 10 times on single leaves of *S. tuberosum.*

### 2.4 Detection of Freezing Exotherms by IDTA

For more detailed analysis of the spatio-temporal freezing behavior of leaves of *S. tuberosum,* infrared differential thermal analysis (IDTA; [10–12]) was applied. In contrast to DTA, IDTA allows for the determination of the location of ice nucleation, ice propagation but also supercooling. A digital infrared camera (ThermaCAM S60, FLIR Systems, Danderyd, Sweden) was used for thermal recording. The differential sequences were obtained by subtraction of images. Subtraction and analysis was conducted with the software ThermaCAM Researcher Pro (version2.10, FLIR Systems, Danderyd, Sweden). Experiments were repeated three times with single leaves.

### 2.5 Ice Susceptibility Test

To check whether leaves were ice susceptible or ice tolerant, leaves were exposed during the freezing treatment to subzero temperatures at −2.5 °C ± 0.2 SE for 2 h and were either ice inoculated or kept in the supercooled state. Experiments were conducted in a freezer with an acrylic glass lid, which allowed for the monitoring of the leaves throughout the whole freezing procedure from the outside. When potato leaves freeze, they typically slightly bend. In the artificially ice inoculated leaves (N = 6), ice nucleation was triggered via the cut surface of the leaf petiole using the following experimental setting: The leaf petiole was wrapped in moist cotton wool that was additionally soaked with a suspension of INA bacteria. The leaves that were intended to stay supercooled received no such treatment (N = 14).

### 2.6 Viability Assessment

Right after the low temperature treatment, frost injured leaves were highly fragile and got limp. Slight blackish discolorations appeared within hours. Leaves were kept in plastic bags on wet paper towels at room temperature for full development of frost damage. Final leaf viability was visually assessed 4 d after the freezing treatment. Intense blackish discoloration of the leaf blade indicated frost damage.

## 3 Results

**Figure 1:**
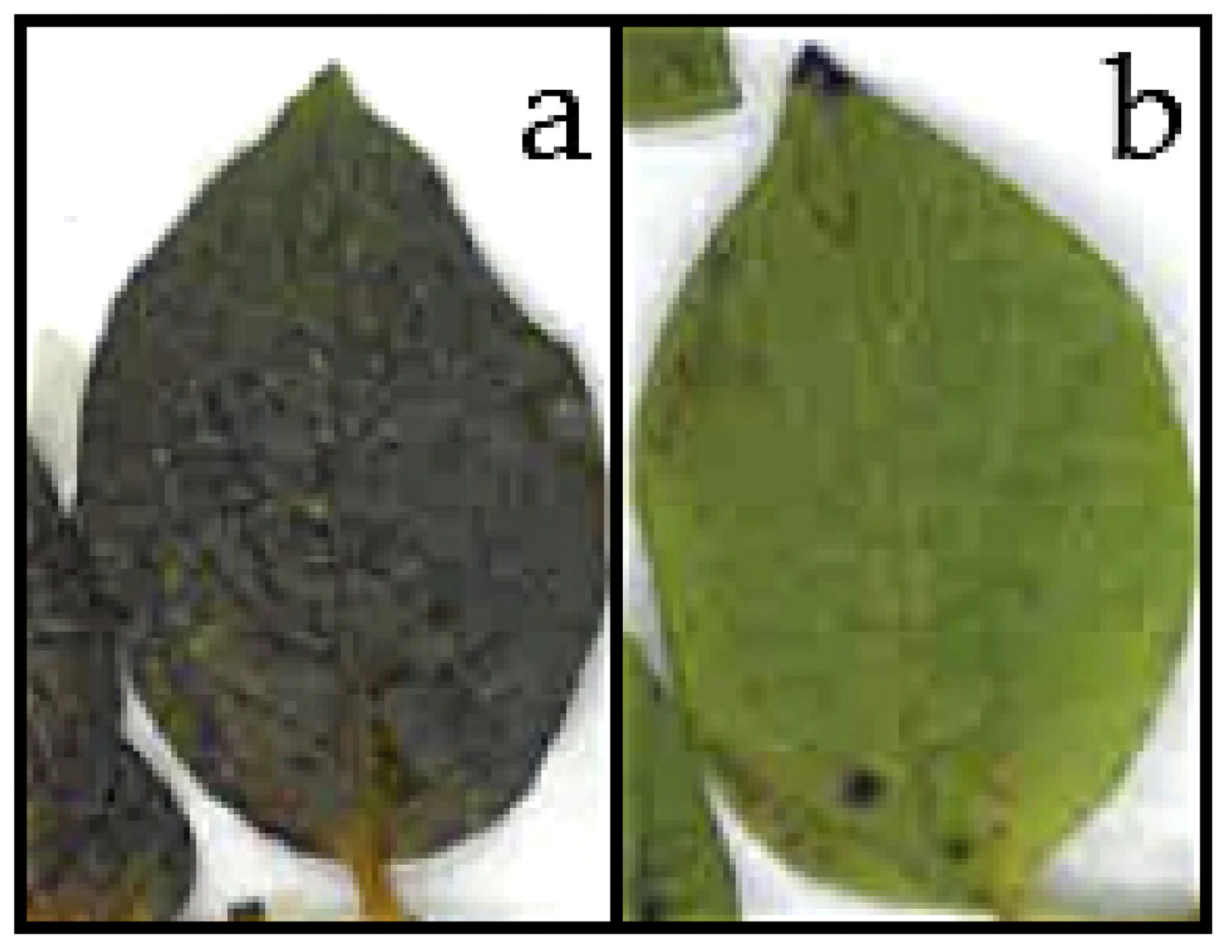
Leaflets of *S. tuberosum* leaves subjected to a controlled freezing treatment down to −3.0 °C either **(a)** inoculated with ice or **(b)** kept supercooled. Digital images were taken 4 d after the low temperature treatment.

Leaves of *S. tuberosum* were subjected to controlled freezing experiments down to −3.0 °C. They were either inoculated with ice or not. In half of the leaves ice nucleation was triggered, the other half remained supercooled. While the ice nucleated leaves showed tissue infiltrations and were limp, the supercooled leaves seemed visually unaltered after the low temperature treatment. 4 d afterwards, frost injuries manifested as blackish discolorations in leaves that had been ice nucleated (Figure 1a). Leaves that were exposed to freezing temperatures of −3.0 °C without ice nucleation showed no symptoms of frost injury on the leaf blade (Figure 1b).

**Figure 2:**
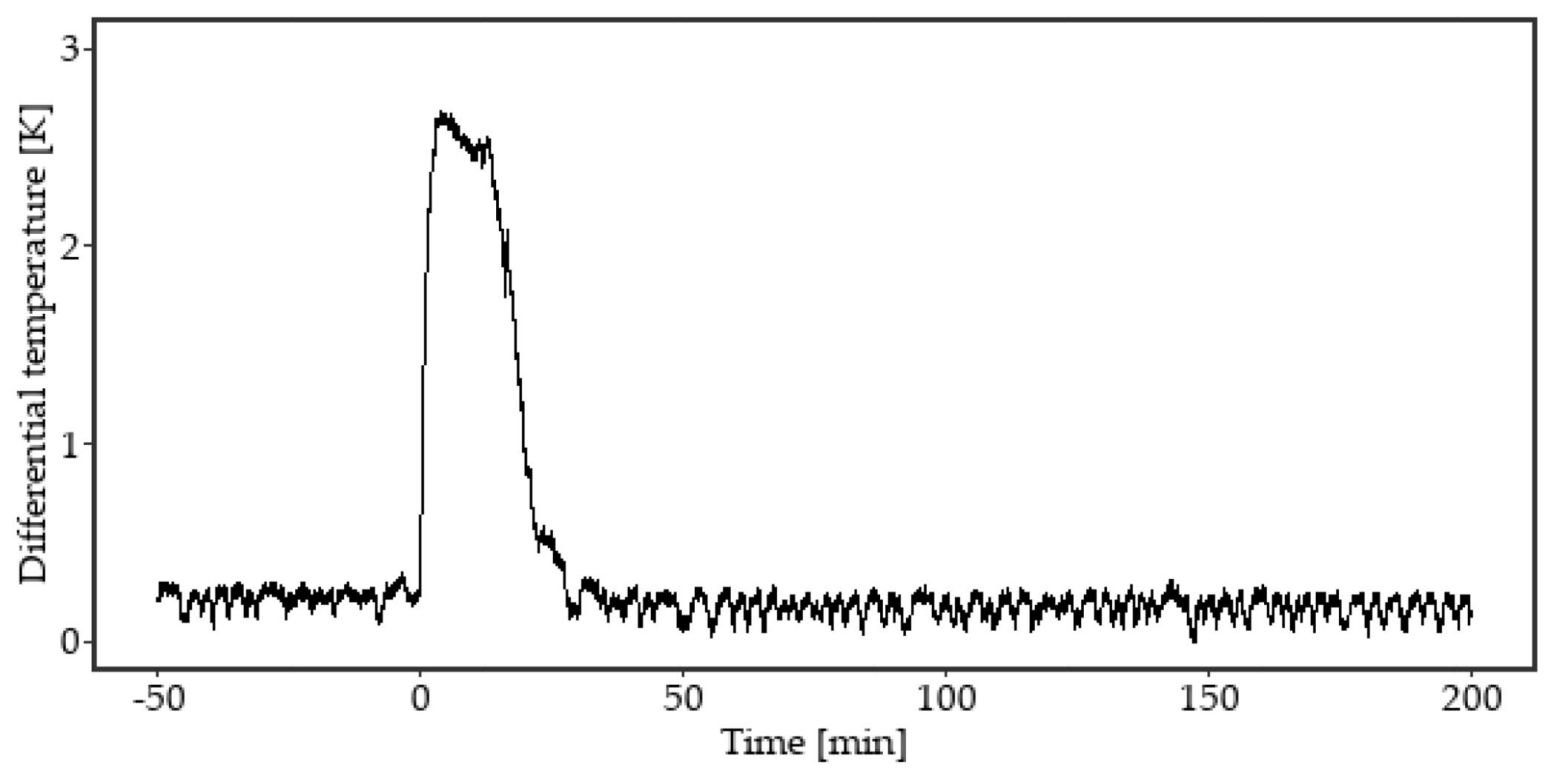
The DTA (differential thermal analysis) plot determined on leaves of *S. tuberosum* during a controlled freezing treatment at a rate of −3 K·h^−1^ showed a single significant freezing exotherm. Minute zero indicated the starting point of the freezing event.

When freezing of leaves of *S. tuberosum* is monitored by DTA, typically one pronounced freezing exotherm is recorded (Figure 2). Under the experimental conditions and with proper thermal insulation of the leaves, the release of heat during freezing lasted for approximately 20 min and exceeded 2.5 K.

**Figure 3:**
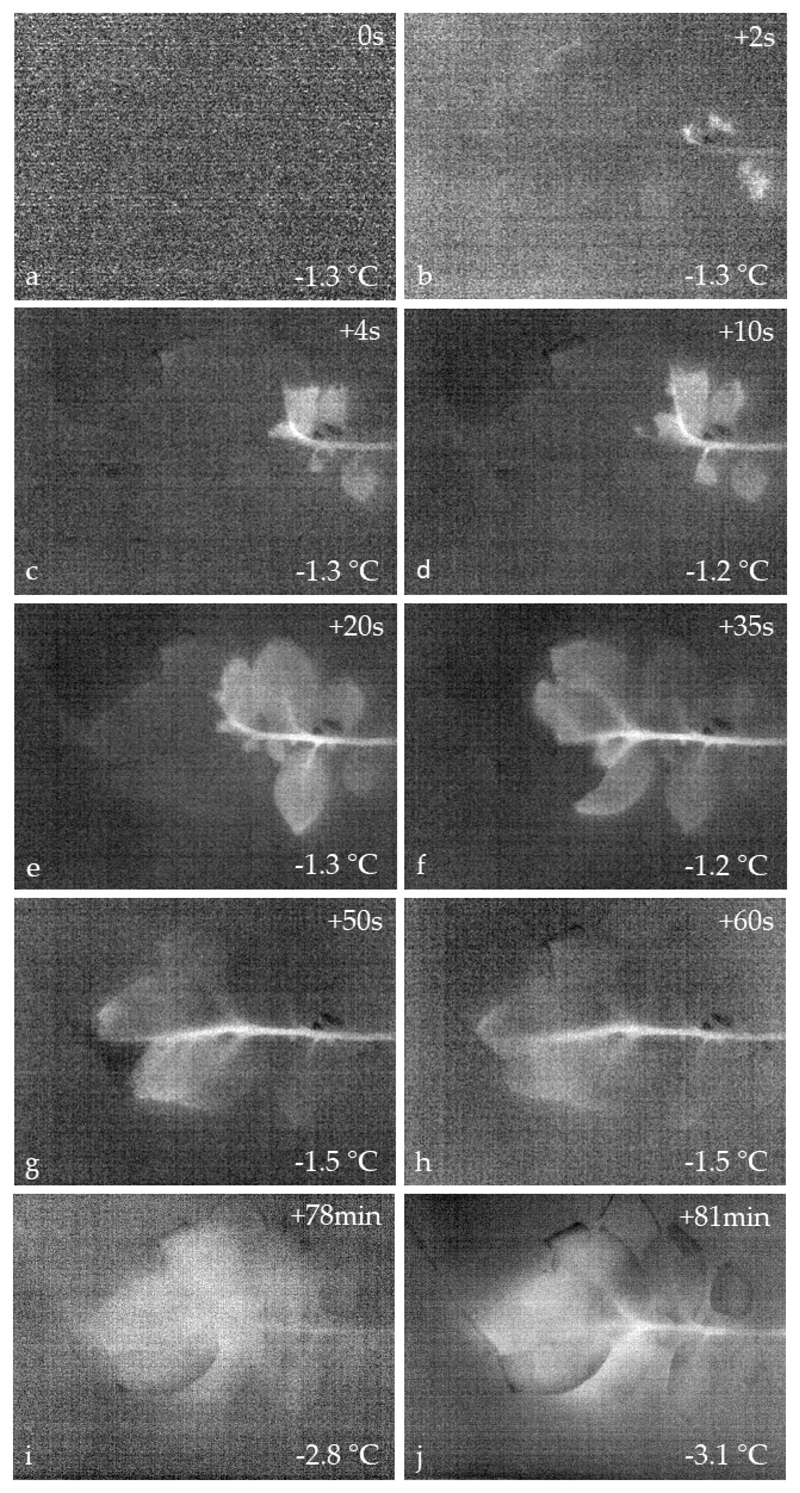
IDTA (infrared differential thermal analysis) images obtained on a leaf of *S. tuberosum* during a controlled freezing treatment at a cooling rate of 3 K·h^−1^. Whitening indicates heat released during freezing processes. The time series **(a–h)** shows the spatial spreading of ice after ice nucleation in the leaf petiole over the entire leaf blade during the first freezing event (Video S1). The freezing of this first exotherm lasted approximately 1 min. Much later **(i–j)** a second freezing event could be detected (Video S2). Time since initial ice nucleation is given in s/min at the top right corner. Leaf temperatures during freezing are indicated in the bottom right corner.

In comparison to DTA, IDTA revealed significantly more details during freezing of the *S. tuberosum* leaves (Figure 3; Video S1; Video S2). Particularly, the spatio-temporal pattern of freezing is visualized. In IDTA, the temperature pattern immediately before the onset of freezing is subtracted from all the subsequent following images. Therefore, the initial differential image showed a homogenous temperature distribution (Figure 3a). The initial ice wave started from the petiole where ice nucleation occurred under the experimental conditions at −1.3 °C (Figure 3b). Rapid ice propagation captured the whole leaf blade within 60 s (Figure 3b–h). The spreading of ice occurred via the leaf vascular system, which was immediately followed by freezing throughout the blade. After 78 min a second freezing event on the leaves was recorded by IDTA (Figure 3i–j). This freezing process differed from the first exothermic event. The freezing pattern was diffuse all over the leaf blade without a distinct starting point. The exothermic response lasted distinctly longer than the first rapid event. After controlled thawing, the leaves were inspected for frost injuries. As expected, after such treatments the whole leaf blades were frost killed.

**Figure 4:**
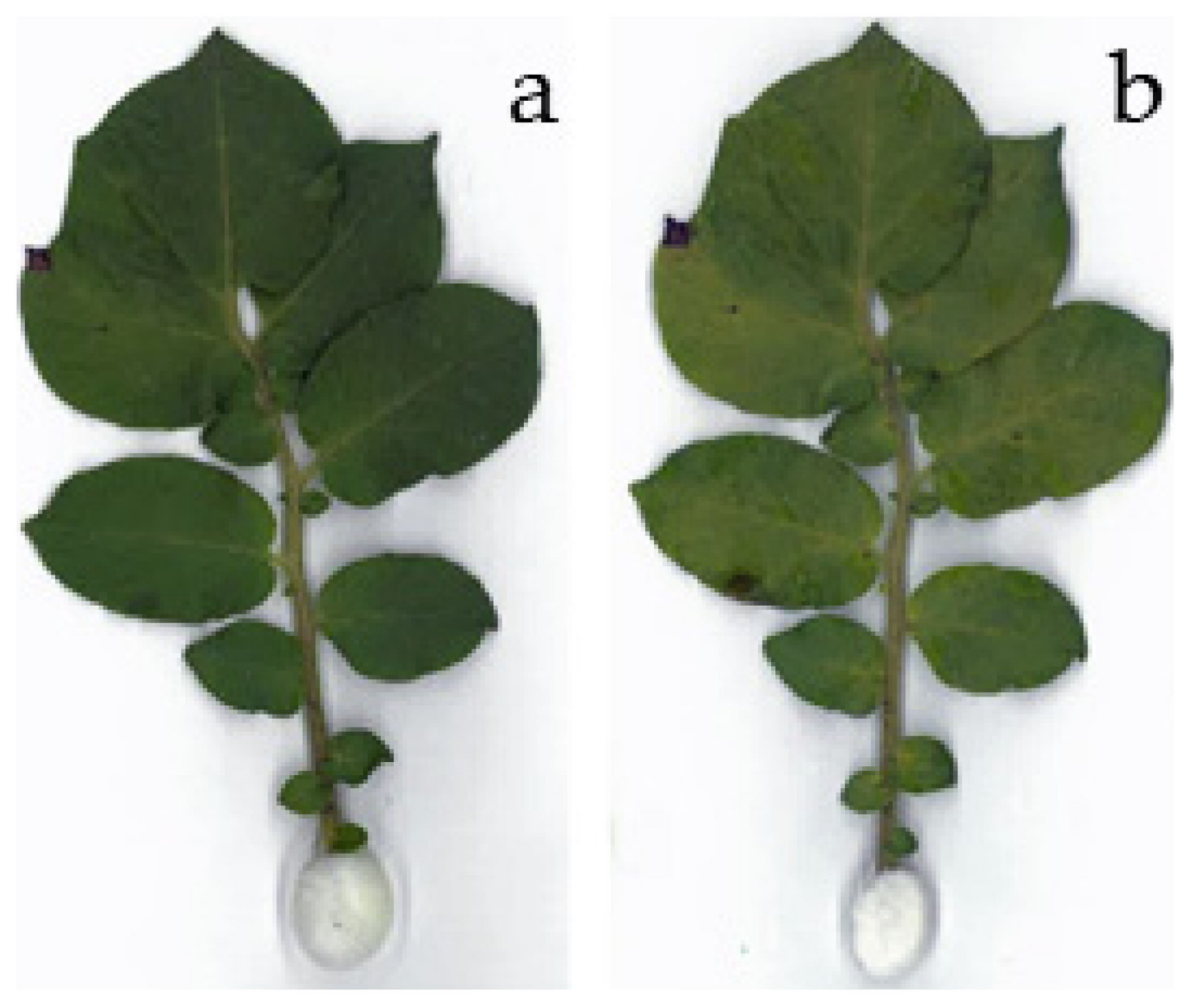
A leaf of *S. tuberosum* **(a)** before and **(b)** after a controlled freezing treatment down to −2.7 °C. The leaf was rewarmed immediately after the first ice wave (as monitored by IDTA) and hence the second freezing was prevented. The digital image after freezing was taken 4 d after the low temperature treatment.

With respect to the results of the ice susceptibility test (see Figure 1), and IDTA results, we intended to test the effect of the first freezing event detected in IDTA on cell viability. For this, leaves were monitored by infrared thermography during cooling at a rate of 3 K·h^−1^. Immediately after the first rapid freezing event (triggered by INA bacteria) the leaves were rewarmed at moderate rates to non-freezing temperatures. Despite ice formation, the leaves did not show any symptoms of frost damage 4 d after the freezing treatment (Figure 4). This was except for a slight change in color due to the onset of leaf senescence that induced chlorophyll degradation.

## 4 Discussion

The use of IDTA under controlled freezing conditions gave new insights into the freezing process of potato leaves and revealed two distinct freezing events. The first freezing event was also detectable by DTA. The average rise in temperature during this first freezing event was 2.5 K. The freezing pattern with initial spread through the vascular system and from there in to the leaf blade and the duration of the overall freezing process were all quite similar to the earlier findings for potato by thermal imaging [27]. Infrared equipment has significantly improved since this early thermal imaging study on potato freezing which might explain why the second exotherm found in the current IDTA study was not detected [27]. Technical advancements in infrared thermography during the last decades nowadays provide much more detailed insights [19]. Leaves removed from the freezing test being frozen or supercooled at −2.5 °C ± 0.2 SE, respectively, showed differential damage symptoms with the frozen leaves becoming completely water soaked and having a darkened appearance and the unfrozen leaves remaining quite healthy. This would suggest ice susceptibility of the leaves. Similar observations have been reported earlier [27]. This clearly demonstrates that frost injury has something to do with the formation of ice in the leaves and as suggested earlier might be an important point for the development of crop frost protection strategies [27].

Strikingly, when leaves were rewarmed after the first freezing exotherm, they did not show any symptoms of frost injury. This suggests that the first freezing event is not lethal. There is some indication in the literature that potato leaves might tolerate freezing [25]. In a thermal imaging study, it was observed that if potato plants were removed from the freezing chamber within 5 min of freezing then injury was limited while irreversible frost damage occurred to plants within 10 min of shoot freezing [27]. This already indicated that the after effects of this ice formation causes frost injuries. A dramatic increase in frost injury of potato leaves (*S. acaule, S. commersonii*) was observed when ice was initiated at about −4 °C as compared to similar leaf tissue frozen to the same freezing temperature but with ice, which was initiated at about −1 ° C [29]. This may also indicate that freezing may be tolerated when ice nucleation occurs at a moderate freezing temperature.

Our findings support that *S. tuberosum* leaves tolerate ice, even though only for a short time period (10–78 min). IDTA reveals that ice initially propagates via the vascular tissue, but at the same time ice captures the whole leaf blade. By IDTA it is not possible to localize where exactly ice forms and propagates in the mesophyll during this initial freezing event. The observed freezing pattern could be extra-tissue freezing [20] where ice accumulates primarily in spaces outside the tissue, and a little in the intercellular space within the tissue. Unfortunately, by microscopic inspection of dissected frozen leaves extracellular/extra-tissue ice masses could not be localized. In this respect, precise localization of ice in leaves after the first freezing event could yield valuable insights about freezing resistance mechanisms.

When leaves remained exposed after the first freezing exotherm under the experimental conditions a second freezing event could be detected. This freezing process had a diffuse pattern and occurred all over the leaf blade. No distinct starting point of this freezing could be depicted. After this second freezing event when leaves had thawed, they were water soaked and limp, which indicated frost damage. Given that during the first freezing event extra-tissue water has frozen, during this second freezing event remaining supercooled water inside the living cells must have frozen. This suggests an intracellular freezing process. Preliminary cryo-microscopic results give evidence that mesophyll cells of potato do not show a freezing cytorrhysis that is commonly the result of freeze dehydration but freeze intracellularly during the second freezing event [30]. The time span between the two freezing events in potato leaves was usually 10 min but could occasionally be up to 78 min. This may suggest that the second freezing exotherm associated with intracellular freezing is a stochastic event. This is typical of most freezing processes originating from breakdown of supercooling as for instance seen in xylem parenchyma cells [13] or supercooling buds [16–18].

*S. tuberosum* is unable to frost harden and gets frost killed around −3 °C [25]. None of a number of commonly cultivated cultivars of *S. tuberosum* examined could survive below −2.5 °C [31]. Our results show that ice formation (first freezing event) per se is not injurious, but consequent processes, i.e., a second freezing exotherm, is responsible for frost damage. Ice nucleation temperature may be more relevant for frost survival than actual frost severity. Still, under field conditions, in leaf and stem tissue of potato ice nucleates at −0.5 to −3.0 °C [21,26] being a stochastic event. Thus, control of ice nucleation as suggested earlier [27] must be considered a key for frost protection of potato.

## Supplementary Material

The following are available online at http://www.mdpi.com/2076-3417/9/5/819/s1. Video S1: First exothermic event: IDTA sequence obtained on a leaf of *S. tuberosum* during a controlled freezing treatment at a cooling rate of 3 K·h^−1^. Whitening shows the spreading of ice after ice nucleation in the leaf petiole over the entire leaf blade. This first exotherm lasted approximately 1 min (playback speed 4x), Video S2: Second exothermic event: IDTA sequence obtained on a leaf of *S. tuberosum* during a controlled freezing treatment at a cooling rate of 3 K·h^−1^. The second freezing event is diffuse without a distinct starting point (playback speed 4x). The video depicts only a part of the second freezing event.

Supplementary materials
